# Synergistic Effects of Mannan Oligosaccharides and Onion Peels on In Vitro Batch Culture Fermentation of High Concentrate and Forage Diets

**DOI:** 10.3390/ani14223180

**Published:** 2024-11-06

**Authors:** Lydia K. Olagunju, Oludotun O. Adelusi, Peter A. Dele, Yasmine Shaw, Rosetta M. Brice, Oluteru E. Orimaye, Jorge A. Villarreal-González, Hye Won Kang, Ahmed E. Kholif, Uchenna Y. Anele

**Affiliations:** 1 Department of Animal Sciences, North Carolina Agricultural and Technical State University, 1601 East Market Street, Greensboro, NC 27411, USA; lkolagunju@aggies.ncat.edu (L.K.O.); ooadelusi@aggies.ncat.edu (O.O.A.); delepa@funaab.edu.ng (P.A.D.); yashaw@aggies.ncat.edu (Y.S.); rosettab101@gmail.com (R.M.B.); oeorimaye@aggies.ncat.edu (O.E.O.); villagon7@gmail.com (J.A.V.-G.); aekholif@ncat.edu (A.E.K.); 2 Department of Family and Consumer Sciences, North Carolina Agricultural and Technical State University, 1601 East Market Street, Greensboro, NC 27411, USA; hkang@ncat.edu

**Keywords:** onion peel, mannan oligosaccharide, gas production, fibers, volatile fatty acids

## Abstract

The current study evaluated the effect of combining mannan oligosaccharide (MOS) and onion peel (OP) on ruminal in vitro total gas (GP), greenhouse gas emissions, dry matter and fiber fraction digestibility, partitioning factor (PF_24_; mg degradable DM per mL gas), microbial mass, and volatile fatty acids using two dietary substrates: high forage (HF) and high concentrate (HC) diets. The study was arranged as a 2 × 2 × 6 factorial design with two dietary substrates, two time points (6 and 24 h), and six treatments. The treatments included a control group with no MOS or OP administration and groups administered with 2% of a mixture containing MOS and OP in the following ratios: 1:0 (MOS), 0:1 (OP), 1:1 (MOS:OP), 1:2 (MOS:2OP), and 1:3 (MOS:3OP). No significant diet × treatment interactions were observed for any of the measured parameters. However, treatments decreased (*p* < 0.05) the undegraded portion of HC, and treatment × substrate interactions were significant (*p* < 0.05) for PF_24_ and microbial mass. The treatments in the HC diet produced higher GP (*p* < 0.001) at 6 h compared to the treatments in the HF diet. Administration of MOS:2OP to the HC diet increased GP at 24 h of incubation, while the lowest GP was observed with the OP in the HF diet. The administration of MOS, OP, and MOS:2OP to the HC diet decreased methane production at 24 h of incubation. Additionally, MOS:2OP and MOS:3OP increased (*p* < 0.001) degradable acid detergent fiber (*d*ADF) in the HC diet at 6 h of incubation. Both OP and MOS:3OP decreased the degradability of acid detergent lignin in the HC diet (*p* < 0.001). The OP also resulted in the lowest DM disappearance (*p* < 0.001) at 24 h of incubation in the HF diet, while the MOS:3OP had the highest *d*ADF. At the end of incubation, the highest productions of total volatile fatty acids and acetate were observed (*p* = 0.002) with the MOS:OP administration in the HC diet, whereas the lowest values were observed with MOS and OP administration to the HF diet. The inclusion of mannan oligosaccharide and onion peel combinations as additives improved substrate (HC and HF) fermentation, leading to higher GP and volatile fatty production, and modulated fiber degradability by improving the breakdown of acid detergent fiber and acid detergent lignin.

## Simple Summary

Natural bioactive compounds with high potential to enhance performance of animals are required to provide strategies to advance animal production. The current study evaluated synergistic effects of mannan oligosaccharide (MOS) and onion peel (OP) in two dairy dietary substrates. Results showed improvement in fermentation parameters and modulation of fiber degradability. Combining MOS and OP was better than their individual administration.

## 1. Introduction

Microbial fermentation of feed in the gastrointestinal tract known as enteric fermentation is the primary source of methane (CH_4_) emissions in ruminants. It is the major agricultural source of CH_4_ emission, with dairy, beef, and sheep contributing 75% of global greenhouse gases emissions of agricultural origin [1]. Since these emissions are associated with a 2–15% loss of dietary potential energy, they also contribute to a reduction in productivity [2]. Several strategies have been assessed to reduce CH_4_ and other greenhouse gases from ruminants. These strategies include the administration of individual or essential oil blends, probiotics, prebiotics (e.g., oligosaccharide), algae, and plant extract rich in biologically active components, yielding mixed results [3,4,5,6,7].

Mannan oligosaccharide (MOS) are prebiotics made up of complex carbohydrate molecules derived from the outer cell wall of *Saccharomyces cerevisiae.* They exhibit stability during feed processing and granulation, maintaining their structural and functional integrity. Mannan oligosaccharide has been recognized as a potential substitute to replace antibiotic additives, and it can improve livestock growth performance, intestinal microflora, antioxidant capacity, and immune function [8]. It can improve animal productivity through better gastrointestinal and ruminal health [9]. MOS can regulate gut microflora in ruminants by maintaining the rumen environment and normal rumen fermentation [10]. Zheng et al. [11] stated that MOS at 2.0% decreased in vitro and in vivo carbon dioxide (CO_2_) and CH_4_ productions.

Onion peel provides plant-derived bioactive compounds (e.g., allicin and kaempferol) that have potent antimicrobial activities which can alter fermentation processes due to their content of chemical constituents and functional groups such as terpenoids, phenolics, flavonoids, flavanols, and phenols [1]. These plant complexes have aromatic lipophilic characteristics capable of binding with rumen metabolites to elicit diverse degradation effects. The presence of such active compounds may alter ruminal fermentation and decrease CH_4_ and other greenhouse gases emission and enhance nutrient degradability [12]. Eom et al. [13] observed that extract of OP at 1, 3, 5, 7, or 9% of a timothy hay-based diet did not affect DM degradability, linearly decreased CH_4_ emission and acetate (C_2_) concentration.

Therefore, it is hypothesized that supplementation of MOS and onion peels (OP) combinations into diets formulated for dairy cattle will work synergistically to decrease greenhouse gases emission, while at least maintaining ruminal fermentation and nutrient digestibility through utilizing the easily fermentable substrate in MOS and bioactive components in OP. Accordingly, the objectives of the present study were to evaluate the effects of MOS and OP supplementation on in vitro nutrient disappearance, and greenhouse gases and gas production of high forage (HF) and high concentrate (HC) diets. The concentrations of crude protein (CP) and fibers were the criteria utilized to determine the difference between HC and HF diets.

## 2. Materials and Methods

### 2.1. Dietary Substrates

Table 1 shows the chemical composition of the two diets, high forage (HF) and high concentrate (HC), used in the present study as previously reported by Brice et al. [7]. The HC consists of grain products, processed grain byproducts, plant protein products, roughage products, molasses products, and multi-vitamins and minerals supplements.

### 2.2. Animal Care and Feeding

Approval (protocol # LA21-009) for the cannulated dairy cows was received from the Institutional Animal Care and Use Committee (IACUC) at North Carolina Agricultural and Technical State University.

### 2.3. Experimental Design

The experiment employed a 2 × 2 × 6 factorial design with two separate runs. The treatments were controlled without MOS or OP administration (Control without additives), or administered with 2% of the diet as a mixture containing MOS and OP at 1:0 (MOS), 0:1 (OP), 1:1 (MOS:OP), 1:2 (MOS:2OP), and 1:3 (MOS:3OP). Four laboratory replicates were prepared for each treatment across the different time periods. The total gas production, in vitro dry matter digestibility (IVDMD), greenhouse gas emissions and efficiency of microbial population (PF_24_, partitioning factor) of the treatments were evaluated.

### 2.4. Sample Preparation

The yellow onion variety (*Allium cepa* L.) was used in this experiment. The peels were cleaned, air-dried, and ground with a Retsch miller (model SM 100; Retsch GmbH, Haan, Germany) using a 2-mm sieve. Five treatments and a control with approximately 0.5 g each were measured using an analytical scale (model VWR-224AC; VWR International, Radnor, PA, USA) as previously described by Brice et al. [7] but four replicates instead of three were used in the present study. For nutrient digestibility, a second set of samples was used.

### 2.5. In Vitro Batch Culture

The batch culture study followed the detailed procedures described by Anele et al. [14]. Rumen fluid was sampled from two cannulated Holstein cows, which were maintained on a grass pasture mix and fed on the same diets as shown in Table 1. The inoculum was mixed with artificial saliva [15] at a 1:3 ratio (15 mL of inoculum: 45 mL of artificial saliva) and incubated at 39 °C for 6 and 24 h. At each time point, accumulated headspace gas pressure was measured using a pressure transducer to prevent gas build-up that could inhibit fermentation. Gas production in all bottles at each time point was calculated using a regression equation derived from the relationship between gas volume and pressure: V = 4.974 × *p* + 0.171 (n = 500; R^2^ = 0.98; where: V is a gas volume (mL); *p* is the measured pressured (psi). Blanks were included to account for gas production from the buffered inoculum, and corrected gas pressure values were used to estimate the gas production [16].

### 2.6. Estimation of Greenhouse Gases

Concentrations of ammonia (NH_3_), CO_2_, hydrogen sulfide (H_2_S), and CH_4_ concentrations were measured using a portable gas analyzer (Biogas 5000, Landtec, Dexter, MI, USA), which was calibrated according to the manufacturer’s instruction. A sample of gas was drawn into the analyzer using a 22 mm gauge needle attached to the end of the inlet Tygon tube. After each sampling, the gas analyzer unit was purged to remove any residual gas from the previous sampling by flushing fresh air between measurements.

### 2.7. In Vitro Dry Matter Digestibility

After taking gas readings, the Ankom bags (Ankom Technology, Macedon, NY, USA) were taken out of the bottles, rinsed, and dried in a 55 °C oven for 48 h. The in vitro apparent degradable dry matter (IVADDM) and in vitro true degradable dry matter (IVTDDM) were estimated following the methods outlined by Anele et al. [17]. The disappearances of DM (*d*DM), NDF (*d*NDF), ADF (*d*ADF), and ADL (*d*ADL) were calculated by subtracting the residual weight of the substrate from the initial weight of the substrate.

### 2.8. Chemical Analysis

The diets were analyzed for DM (#930.15), N (#954.01), and ether extract (EE; #920.39) following AOAC methods [18]. The equipment used to estimate the N, EE, and fiber components have been previously described by Brice et al. [7]. Neutral detergent fiber (NDF) and acid detergent fiber (ADF) were analyzed as described by Van Soest et al. [19], utilizing a heat-stable α-amylase with sodium sulfite and according to AOAC [18] (method 973.18), respectively. Acid detergent lignin (ADL) was analyzed by soaking samples in a concentrated sulfuric acid base, following the analytical methods established by Ankom Technologies (Ankom Technology, Macedon, NY, USA).

### 2.9. Microbial Mass Estimation

Microbial mass (MM) was estimated following the protocol outlined by Olagunju et al. [20], as referenced previously by Blümmel and Lebzien [21]. The PF_24_ was estimated as the ratio of mg of substrate truly degraded/mL of gas produced [22], following the procedure described by Olagunju et al. [20].

### 2.10. Volatile Fatty Acid

The volatile fatty acid (VFA) concentration was analyzed using a gas chromatography as described by Olagunju et al. [20].

### 2.11. Statistical Analysis

Data generated were analyzed using the GLM procedure of SAS 9.4 (SAS Inst., Inc., Cary, NC, USA) in a 2 × 2 × 6 factorial arrangement. Treatment effects and interactions were examined by using the probability of difference (PDIFF) option of the least square means statement in the MIXED procedure of SAS. The means were separated using Duncan’s multiple comparisons test at *p* < 0.05.

## 3. Results

### 3.1. In Vitro Fermentation

No significant diet × treatment interactions and treatment effects were observed for IVADDM, IVTDDM, PF_24_, undegraded residuals and MM (Table 2). However, diet type was significant (*p* < 0.05) for all the parameters. The HC diet had higher IVADDM, IVTDDM, PF_24_ and MM, with lower values for the undegraded residuals.

### 3.2. Greenhouse Gases Production

No significant diet × treatment interactions were observed with total gas and greenhouse gas production (Table 3). At 6 h of incubation, treatments did not affect the concentrations of NH_3_ and H_2_S. Treatments did not affect the amounts of produced gases; however, the treatments in the HC diet produced higher gas (*p* < 0.001) compared to the treatments in the HF diet. The highest CH_4_ production was observed with the MOS:3OP treatment to the HC diet (*p* < 0.001), while MOS:OP, MOS:2OP, and MOS:3OP treatments in the HF had the lowest CH_4_ production. The MOS:OP and MOS:3OP treatments increased CO_2_ production from the HC diet; however, the administration of all treatments to the HF increased CO_2_ production compared to that with no treatments.

At 24 h of incubation, treatments did not affect CO_2_, NH_3_ and H_2_S (Table 3). The highest gas production (*p* = 0.002) was observed with the MOS:2OP diet, while the lowest gas production was observed with the OP treatments to the HF diet. The administration of MOS, OP and MOS:2OP to the HC diet decreased CH_4_ production, while the administration of OP to the HF diet resulted in the lowest CH_4_ production compared to all treatments.

### 3.3. Dry Matter and Fiber Disappearance

No significant diet × treatment interactions were observed for nutrient disappearance (Table 4). At 6 h of incubation, *d*NDF was higher (*p* < 0.001) in the HF compared to the HC diet with no treatment effect noted for the diets. Both MOS:2OP and MOS:3OP treatments increased (*p* < 0.001) *d*ADF in the HC diet compared to the control, while in the HF diet, the OP and MOS:OP treatments decreased *d*ADF compared to the MOS:3OP which had the highest *d*ADF value (*p* < 0.001). Compared to the control, both OP and MOS:3OP treatments decreased *d*ADL in the HC diet (*p* < 0.001), with no effect for the other treatments. With the HF diet, no significant effects were observed with the treatments on *d*ADL; however, MOS:2OP and MOS:3OP had the lowest *d*ADL in all treatments.

At 24 h of incubation, treatments did not affect *d*DM of the HC or HC diets; however, the OP treatment had the lowest *d*DM (*p* < 0.001) compared to all treatments (Table 4). Treatments did not affect the *d*NDF within each diet; however, the HF diet had higher *d*NDF compared to the HC diet. The HC diet had higher *d*ADF and lower *d*ADL compared to the HC diet (*p* < 0.001). The MOS:3OP treatment had the highest *d*ADF of the HC and HF diets.

### 3.4. Volatile Fatty Acids Production

No significant diet × treatment interactions were observed for total or individual VFA (Table 5). At 6 h of incubation, diets did not affect total or individual VFAs. At 24 h of incubation, treatments did not affect the concentrations of propionate (C_3_), C_2_:C_3_ ratio, isobutyrate (iso-C_4_), and isovalerate (iso-C_5_). The highest production of total VFA and C_2_ were observed (*p* = 0.002) with the MOS:OP administration to the HC diet while the lowest values were observed with MOS and OP treatments to the HF diet. The administration MOS:OP and MOS:3OP to the HC increased butyrate (C_4_) (*p* = 0.003) and valerate (C_5_) (*p* < 0.001) concentrations, while the OP and MOS administration to the HF diet resulted in the lowest C_4_ and C_5_ (*p* < 0.001) concentrations.

## 4. Discussion

The absence of diet × treatment interactions with all measured parameters are diet and treatment dependent, and this shows that each treatment affected the diets differently.

### 4.1. In Vitro Fermentation

Expectedly, the HC diet had higher IVADDM, IVTDDM, PF_24_, and MM values and lower undegraded residual value compared to the HF diet. This indicates that the HC diet was more extensively degraded than the HF, which could be attributed to higher CP and lower fiber concentrations in the HC diet compared to the HF diet. Variations in the undegraded portions of the substrates would be related to their chemical composition with lower values noted for the HC diet due to its lower fiber concentration. These results may be related to the availability of more nutrients for microbial activity since the PF_24_ value has a positive relationship with MM. In an in vitro experiment, Eom et al. [13] reported that the inclusion onion extract from 1 to 9% did not affect ruminal microbial growth rate, which may partially explain the present results. The weak effects of treatments on the parameters may be related to the inclusion level and incubation period. Increasing the incubation period may give the ruminal microbes time to adapt to the additives and enhance their effects on nutrient degradability.

### 4.2. Greenhouse Gases Production

The chemical composition of a diet, especially its contents of CP and fibers is one of the main factors effecting the diet’s fermentability [23]. Morsy et al. [24] stated that increasing fiber and decreasing CP concentrations will increase GP and lag time. In the present study, the HF diet had 6.72% CP vs. 16.6% in the HC diet; however, the HF diet had 44.7% NDF vs. 32% in the HC diet. The fermentation of protein gives a relatively small amount of gas compared to the fermentation of carbohydrates [25]. This observation is not consistent with the present results or the results of Kholif et al. [23] who noted that an increase in fiber concentration in the diet resulted in a decrease in the total GP.

Initially, the HC diet produced more gas compared to the HF diet, indicating a shorter lag time of GP with the HC diet, and could be related to the higher concentrations of fermentable materials in the HC compared to the HF diet [26]. Previous studies showed that HC diet produces more gas than HF diets [23,27]. Conversely, at the end of incubation there were no differences between the HC and HF diets. This may be because the ruminal microbes quickly utilized the available carbohydrates to produce gases during the first hours of the incubation.

At 6 h of incubation, treatments did not affect the amounts of produced gases, indicating that the ruminal microbes needed more time to be positively or negatively affected by the administered additives. However, the administration of MOS:2OP to the HC diet had the highest GP at the end of incubation. Interestingly, the individual MOS and OP did not affect GP, which confirms the synergism between them, and may be related to the presence of easily available carbohydrates in the HC diets which may mask the negative effects of OP as observed with the administration of OP to the HF diet which lowered the GP at the end of incubation. Such results may be related to the content of kaempferol (major flavonoid in onion) which is associated with reduced total GP in ruminants due to its antimicrobial effects on ruminal microbes [28]. Ruminal microbial activity is closely correlated with total GP, which in turn is related to DM degradability [28]. As seen in previous studies, the effect of MOS administration on GP is weak [11]; however, Wang et al. [29] reported cumulative GP increases linearly with increasing doses of oligosaccharides administration.

Reducing greenhouse gases could reduce energy loss and greenhouse gas emissions of ruminants, which could contribute to improved animal productivity. The administration of MOS:3OP to the HC diet increased CH_4_ production, indicating that increasing the level of OP in the additive mixture increases CH_4_ output. In contrast, the administration of MOS, OP, and MOS:2OP to the HC diet decreased CH_4_ production at the end of incubation, which may be related to the presence of easily fermentable carbohydrates like MOS which has been associated with lower CH_4_ production due to the inefficient conversion of potentially available energy [30]. The presence of prebiotics like MOS has the potential to selectively encourage the growth and activity of some ruminal bacteria (e.g., cellulolytic bacteria), thereby enhancing fiber digestion and promoting overall rumen fermentation [31].

The administration of MOS:OP, MOS:2OP, and MOS:3OP to the HF decreased CH_4_ production, which is an important result when feeding animals on HF diet, that are known to produce more CH_4_ compared to the HC. This is a positive development for the use of a HF diet for ruminant feeding, which is relatively cheaper compared to a higher quality diet. These results (gas and CH_4_ productions) indicate that the administration of these treatments is recommended for the HF diets compared to the HC diets.

The administration of OP to the HF diet had the lowest CH_4_ production at the end of incubation. Onion peel is rich in allicin which could inhibit thiol enzyme reactivation causing inhibiting methanogenic archaea activity [32,33]. Moreover, the organosulfur compounds in OP may inhibit HMG-CoA reductase that catalyzes the synthesis of isoprenoid units in the membranes of methanogenic archaea resulting in inhibiting CH_4_ emission. Moreover, the presence of flavonoid in OP confirms the CH_4_-suppressing capacity of OP [28]. Eom et al. [13] stated that the extract of onion linearly decreased CH_4_ emission at 12 h incubation. They also reported a reduced methanogenic archaea abundance with onion extracts.

Treatments (only MOS:OP and MOS:3OP with the HC diet) increased CO_2_ production at 6 h of incubation, because of the presence of easily digestible carbohydrates in the HC diet and the support effect of oligosaccharides being transferred into rumen as substrate for microbes’ fermentation which produced more CO_2_. Methanogenesis can use CO_2_ as a substrate in ruminants [34]. Therefore, lowering CH_4_ production may be a reason for increasing CO_2_ accumulation in the incubation bottles. Kim et al. [35] stated that allicin (an active compound in OP) increased production of CO_2_ in the rumen of cow.

The weak effect of treatments on NH_3_ at the end of incubation indicates an unaffected dynamic balance between microorganisms degrading CP and non-protein nitrogen of diet to produce NH_3_-N and using NH_3_-N to synthesize microbial protein in the rumen [36].

### 4.3. Dry Matter and Fiber Disappearance

The HF diet had higher *d*NDF compared to the HC diet at 6 h of incubation, and higher *d*ADL at the end of incubation confirming the fact that HF diets contain more digestive fibers than HC diets. Elghandour et al. [37] reported that diets with high concentrate levels had higher *d*DM compared to those with high forage. This may be related to increased ruminal activity due to more nutrients being available with these rations.

The HF diet had higher *d*NDF and *d*ADF compared to the HC diet at the end of incubation, which could be due to the high fiber concentration in the HF compared to HC diets [38]. Chen et al. [38] stated that the HC diet has lower *d*NDF compared to the HF diet. This effect could be related to the lowered ruminal pH of the HC diet which negatively affects cellulolytic bacteria in the rumen [39].

The weak effect of treatments on nutrient degradability at 6 h and at the end of incubation is consistent with the results of GP. The OP treatment had the lowest *d*DM in all diets at the end of incubation, which may be related to the antimicrobial effects of secondary metabolites in OP. A decreased *d*DM of a diet indicates less digestion, and hence less nutrients from the diet that would be available to animals. Eom et al. [13] observed unaffected DM degradability with the administration of onion extract from 1 to 9%. Onion contains high levels of allicin and kaempferol, which have previously been shown to have antibacterial and antioxidant activities against ruminal bacteria [32,40].

Both MOS:2OP and MOS:3OP enhanced *d*ADF in the HC diet after 6 h of incubation, while MOS:3OP treatment had the highest *d*ADF of the HC and HF diets at the end of incubation. Eom et al. [13] noted increased abundance of cellulolytic bacteria (*Ruminococcus albus*, *Fibrobacter succinogenes*, and *Ruminococcus flavefaciens*) with the addition of 7% onion extract at 24 h incubation. The presence of MOS and OP worked synergistically to selectively proliferate some fiber-degrading microbes and improve microbial population [13,41].

Both the OP and MOS:3OP treatments decreased *d*ADL of the HC and HF diets at 6 h of incubation confirming the antimicrobial properties of OP and its effects on inhibiting fiber degrading microbes [42]. Moreover, the OP and MOS:OP treatments decreased *d*ADF in the HF diet at 6 h of incubation; however, increasing the OP in the additive mixture (i.e., the MOS:3OP) increased *d*ADF of the HF at 6 h of incubation. The antimicrobial effects of secondary metabolites in OP may be the reason for the lowered *d*ADF during the first hours of incubation, while during the last hours of incubation the ruminal microbes became more adapted to their presence. Results of the enhanced *d*ADF confirm the possible synergism between OP and MOS.

### 4.4. Volatile Fatty Acids Production

Generally, the diets had a minimal effect on the concentrations of total and individual VFAs, which was unexpected given that VFA production mainly depends on the nutrient content, particularly structural and nonstructural carbohydrates [43]. It is well established that C_2_ concentration is influenced by fiber concentration and digestibility, while C_3_ is driven by the concentration and digestibility of nonstructural carbohydrates [44]. In the present experiment, the HC diet contained lower levels of OM (93.4% vs. 95.8%), NDF (32% vs. 44.7%) and ADF (13.7% vs. 21.3%) but a higher concentration of NSC (39.2% vs. 38.4) compared to the HF diet. This composition was expected to increase C_3_ and reduce C_2_ production. However, the short incubation period (i.e., 24 h) may have contributed to these observations.

The weak effect of diets on the total or individual VFA at 6 h of incubation is inconsistent with the assumption that the presence of easily available nutrients in the HC diet encouraged the ruminal microbes for growth and activity to degrade nutrients. The weak effect of treatments on C_3_, C_2_:C_3_ ratio, iso-C_4_ and iso-C_5_ concentrations at the end of incubation, and decreasing CH_4_ production at the same time indicate that the OP would reduce CH_4_ emission without affecting feed efficiency in ruminants. It was expected that the individual and combined additives would improve the production of C_3_ as suggested by Uyeno et al. [45] who reported that oligosaccharide improves C_3_ production.

At the end of incubation at 24 h, the administration of MOS:OP to the HC diet increased total VFA and C_2_. Increasing VFA supports from 70 to 80% of animal’s energy needs [46]. The synergy between OP and MOS was clear since the individual treatments did not affect the production of total and individual VFA. The MOS supplementation increased the abundance of hemicellulose-related degrading bacteria species, which resulted in better carbohydrate fermentation in the rumen [47]. Increasing total VFA and GP with oligosaccharides at the same time has been suggested for improving the efficiency of nutrient utilization [29].

Acetate, though leading to less energy availability for the animal, is considered important in milk fat synthesis as it is used in the mammary gland as a substrate for milk fat [48]. The C_2_ is converted into fatty acids, which are then incorporated into triglycerides, the main constituents of milk fat.

At the end of incubation, the administration of MOS and OP to the HF diet decreased total VFA, C_2_, C_4,_ and C_5_ production. The observed lower VFA production was likely a reflection of the lower digestibility indices recorded in the diet. Eom et al. [13] observed a decrease in ruminal C_2_ concentration with the addition of onion extract as a result of a decrease in the abundance of *Fibrobacter succinogenes* at 12 h incubation.

## 5. Conclusions

Results from the present study showed that the inclusion of MOS and OP combination influenced in vitro fermentation parameters of HC and HF diets. The MOS:OP treatment resulted in higher gas production when compared to individual MOS and OP treatments. Greenhouse gas emissions were not affected by the various treatments. While dry matter and neutral detergent fiber disappearance remained unaffected, the treatments enhanced degradability of acid detergent fiber and acid detergent lignin in both diets. Total VFA and molar proportions of butyrate and valerate were increased by the treatments. Acetate proportion decreased with treatment inclusion, while propionate was not affected. We conclude that combination of MOS:OP improved fermentative efficiency by increasing gas production, modulating fiber digestibility, and influencing molar concentration of individual VFA.

## Figures and Tables

**Table 1:** Chemical composition (%, DM basis) of the basal substrate ^1^.

	High Forage	High Concentrate
Dry matter	96.2	95.8
Organic matter	95.8	93.4
Total ash	4.20	6.60
Crude protein	6.72	16.6
Ether extract	5.95	5.60
Nonstructural carbohydrates	38.4	39.2
Neutral detergent fiber	44.7	32.0
Acid detergent fiber	21.3	13.7
Acid detergent lignin	1.67	2.86

^1^ Previously reported by Brice et al. [7].

**Table 2:** Effects of mannan oligosaccharide (MOS), onion peel (OP) and their combinations on digestibility and microbial mass parameters of high forage (HF) and high concentrate (HC) diets.

Diet	Treatment ^1^	IVTDDM (%)	IVADDM (%)	PF_24_	Undegraded Residuals (%)	MM (g/kg DM)
HC	Control	53.6	39.3	2.47	12.0	0.118
	MOS	51.4	38.4	2.40	11.5	0.128
	OP	53.5	39.0	2.45	11.8	0.118
	MOS:OP	52.9	39.5	2.45	11.4	0.126
	MOS:2OP	53.8	39.3	2.40	11.4	0.120
	MOS:3OP	54.7	38.6	2.37	11.1	0.115
HF	Control	50.4	33.3	2.18	16.7	0.081
	MOS	52.3	33.5	2.17	16.1	0.076
	OP	52.3	33.0	2.22	16.4	0.072
	MOS:OP	51.8	34.3	2.17	16.8	0.079
	MOS:2OP	50.7	34.2	2.18	16.4	0.085
	MOS:3OP	51.1	33.6	2.08	16.1	0.082
	Pooled SEM	1.32	0.48	0.043	0.23	0.0065
	*p* value					
	Diet	0.018	<0.001	<0.001	<0.001	<0.001
	Treatment	0.444	0.951	0.150	0.340	0.827
	Diet × Treatment	0.533	0.718	0.893	0.541	0.638

Within each incubation time, means in the same column with different letters differ, *p* < 0.05. *p*-value is the observed significance level of the F-test, SEM = standard error of the mean. ^1^ Diets were incubated without additives (Control treatment) or with the addition of 2% mannan oligosaccharide and onion peel combination at 1:0 (MOS treatment), 0:1 (OP treatment), 1:1 (MOS:OP treatment), 1:2 (MOS:2OP treatment) and 1:3 (MOS:3OP treatment). IVADDM is in vitro apparent degradable dry matter (%); IVTDDM is in vitro true degradable dry matter (%); PF_24_ is partitioning factor (mg degradable DM: mL gas), MM is microbial mass (g/kg DM).

**Table 3:** Effects of mannan oligosaccharide (MOS), onion peel (OP) and their combinations on greenhouse gases produced from high forage (HF) and high concentrate (HC) diets at 6 and 24 h of incubation.

Incubation Time (h)	Diet	Treatment ^1^	GP (mL/g DM)	CH_4_ (mg g/DM)	CO_2_ (mg g/DM)	NH_3_ (mmol/g DM)	H_2_S (mmol/g DM)
6	HC	Control	20.9 ^a^	0.22 ^abcd^	5.36 ^bc^	32.7	119.1
		MOS	20.4 ^a^	0.25 ^abc^	6.05 ^ab^	35.2	141.9
		OP	21.0 ^a^	0.25 ^abc^	6.08 ^ab^	36.5	164.4
		MOS:OP	21.7 ^a^	0.26 ^ab^	6.47 ^a^	34.1	123.8
		MOS:2OP	20.8 ^a^	0.26 ^ab^	6.15 ^ab^	35.3	140.1
		MOS:3OP	22.2 ^a^	0.30 ^a^	7.09 ^a^	36.8	153.2
	HF	Control	14.1 ^b^	0.07 ^e^	1.69 ^d^	11.	35.5
		MOS	14.4 ^b^	0.15 ^de^	4.05 ^bc^	22.2	95.8
		OP	13.1 ^b^	0.15 ^de^	3.77 ^cd^	20.9	76.4
		MOS:OP	13.0 ^b^	0.14 ^cd^	3.68 ^cd^	21.7	80.1
		MOS:2OP	15.8 ^b^	0.17 ^bcd^	4.25 ^bc^	22.9	100.4
		MOS:3OP	14.9 ^b^	0.17 ^bcd^	4.12 ^bc^	22.1	84.5
		SEM	0.82	0.019	0.450	6.20	27.27
		*p* value	<0.001	<0.001	<0.001	0.074	0.057
24 h	HC	Control	158.7 ^ab^	5.99 ^a^	48.5	314.3	1681.8
		MOS	159.9 ^ab^	5.63 ^b^	44.6	321.7	1570.3
		OP	159.4 ^abc^	5.55 ^b^	43.5	325.1	1509.7
		MOS:OP	161.3 ^ab^	5.93 ^a^	50.5	352.7	1593.2
		MOS:2OP	164.6 ^a^	5.57 ^b^	51.6	346.2	1517.0
		MOS:3OP	163.0 ^ab^	6.03 ^a^	51.1	321.8	1571.3
	HF	Control	152.7 ^abc^	5.27 ^b^	39.8	146.8	1223.8
		MOS	154.8 ^abc^	5.61 ^b^	44.1	295.7	1391.1
		OP	148.8 ^d^	4.87 ^c^	39.4	245.9	1285.7
		MOS:OP	158.3 ^abc^	5.79 ^b^	50.3	317.9	1397.3
		MOS:2OP	156.8 ^abc^	5.90 ^ab^	48.6	281.0	1267.6
		MOS:3OP	161.9 ^ab^	5.86 ^ab^	48.2	332.6	1427.1
		SEM	2.26	0.188	3.47	89.14	106.89
		*p* value	0.002	0.039	0.145	0.951	0.069
		Pooled SEM	1.70	0.770	2.48	6.32	78.00
		Pooled *p* value					
		Diet	<0.001	0.579	0.005	0.148	<0.001
		Treatment	<0.001	0.988	<0.001	<0.001	<0.001
		Diet × Treatment	0.456	0.996	0.768	0.936	0.685

Within each incubation time, means in the same column with different letters differ, *p* < 0.05. *p*-value is the observed significance level of the F-test, SEM = standard error of the mean. ^1^ Diets were incubated without additives (Control treatment) or with the addition of 2% mannan oligosaccharide and onion peel combination at 1:0 (MOS treatment), 0:1 (OP treatment), 1:1 (MOS:OP treatment), 1:2 (MOS:2OP treatment) and 1:3 (MOS:3OP treatment).

**Table 4:** Effects of mannan oligosaccharide (MOS), onion peel (OP) and their combinations on dry matter and fiber degradability of high forage (HF) and high concentrate (HC) diets at 6 and 24 h of incubation.

Incubation Time (h)	Diet	Treatment ^1^	*d*DM (% DM)	*d*NDF (% DM)	*d*ADF (% DM)	*d*ADL (% DM)
6	HC	Control	14.8	38.5 ^b^	45.9 ^e^	11.03 ^a^
		MOS	15.2	38.6 ^b^	45.6 ^e^	10.48 ^a^
		OP	14.6	38.6 ^b^	46.8 ^de^	8.82 ^bc^
		MOS:OP	14.1	38.3 ^b^	46.4 ^e^	10.70 ^a^
		MOS:2OP	15.2	37.3 ^b^	49.2 ^cd^	9.70 ^ab^
		MOS:3OP	15.4	37.5 ^b^	51.2 ^c^	8.66 ^bc^
	HF	Control	15.0	51.9 ^a^	56.5 ^ab^	7.26 ^bc^
		MOS	15.3	50.5 ^a^	56.8 ^ab^	7.81 ^bc^
		OP	15.2	51.1 ^a^	56.1 ^b^	7.30 ^bc^
		MOS:OP	14.1	49.6 ^a^	56.2 ^b^	7.78 ^bc^
		MOS:2OP	14.8	49.7 ^a^	58.3 ^ab^	6.52 ^c^
		MOS:3OP	14.5	49.6 ^a^	58.8 ^a^	6.95 ^c^
		SEM	0.84	0.95	0.52	0.51
		*p* value	0.385	<0.001	<0.001	<0.001
24 h	HC	Control	27.3 ^a^	50.1 ^b^	48.3 ^d^	12.86 ^ab^
		MOS	26.8 ^ab^	48.3 ^b^	48.9 ^cd^	13.58 ^a^
		OP	27.5 ^a^	49.9 ^b^	49.2 ^cd^	12.37 ^ab^
		MOS:OP	27.6 ^a^	49.2 ^b^	48.7 ^cd^	12.23 ^ab^
		MOS:2OP	27.0 ^ab^	47.6 ^b^	50.4 ^cd^	12.52 ^ab^
		MOS:3OP	27.6 ^a^	48.4 ^b^	51.3 ^c^	10.65 ^bc^
	HF	Control	24.7 ^b^	64.9 ^a^	57.1 ^ab^	9.39 ^c^
		MOS	25.6 ^ab^	66.2 ^a^	56.6 ^ab^	8.60 ^c^
		OP	24.9 ^b^	66.1 ^a^	58.2 ^ab^	8.79 ^c^
		MOS:OP	25.7 ^ab^	65.2 ^a^	58.6 ^ab^	9.08 ^c^
		MOS:2OP	25.6 ^ab^	65.3 ^a^	58.9 ^ab^	9.07 ^c^
		MOS:3OP	25.6 ^ab^	64.9 ^a^	59.7 ^a^	7.98 ^c^
		SEM	0.50	0.80	0.59	0.59
		*p* value	<0.001	<0.001	<0.001	<0.001
		Pooled SEM	0.50	0.88	0.56	0.550
		Pooled *p* value				
		Diet	<0.001	<0.001	<0.001	<0.001
		Treatment	<0.001	<0.001	<0.001	<0.001
		Diet × Treatment	0.228	0.875	0.188	0.267

Within each incubation time, means in the same column with different superscripts differ, *p* < 0.05. *p*-value is the observed significance level of the F-test, SEM = standard error of the mean. ^1^ Diets were incubated without additives (Control treatment) or with the addition of 2% mannan oligosaccharide and onion peel combination at 1:0 (MOS treatment), 0:1 (OP treatment), 1:1 (MOS:OP treatment), 1:2 (MOS:2OP treatment), and 1:3 (MOS:3OP treatment). *d*DM is dry matter degradability; *d*NDF is neutral detergent fiber degradability; *d*ADF is acid detergent fiber degradability; *d*ADL is acid detergent lignin degradability.

**Table 5:** Effects of mannan oligosaccharide (MOS), onion peel (OP) and their combinations on total and individual volatile fatty acids (mmol/g DM) of high forage (HF) and high concentrate (HC) diets at 6 and 24 h of incubation.

Incubation Time (h)	Diet	Treatment ^1^	Total	C_2_	C_3_	C_2_:C_3_	C_4_	C_5_	Iso-C_4_	Iso-C_5_
6	HC	Control	43.2	29.3	9.02	3.18	3.95	0.68	0.21	0.11
		MOS	46.9	32.4	9.36	3.46	4.07	0.68	0.22	0.11
		OP	47.4	32.7	9.52	3.42	4.16	0.67	0.21	0.11
		MOS:OP	47.1	32.5	9.47	3.42	4.18	0.64	0.22	0.11
		MOS:2OP	45.6	31.5	9.21	3.39	3.98	0.61	0.21	0.11
		MOS:3OP	48.5	33.9	9.46	3.54	4.18	0.68	0.22	0.11
	HF	Control	43.4	29.3	9.11	3.19	3.91	0.72	0.22	0.12
		MOS	46.0	31.5	9.37	3.34	4.06	0.71	0.22	0.12
		OP	45.5	31.2	9.32	3.33	3.97	0.67	0.22	0.12
		MOS:OP	45.4	31.3	9.25	3.38	3.90	0.61	0.21	0.11
		MOS:2OP	46.8	32.4	9.38	3.44	4.00	0.66	0.21	0.11
		MOS:3OP	47.3	32.9	9.39	3.48	4.06	0.67	0.22	0.12
		SEM	3.10	2.313	0.352	0.126	0.330	0.093	0.016	0.008
		*p* value	0.990	0.968	0.998	0.999	0.999	0.999	0.999	0.999
24 h	HC	Control	50.1 ^ab^	34.0 ^ab^	10.40	3.28	4.68 ^abc^	0.70 ^bcd^	0.24	0.12
		MOS	50.7 ^ab^	34.5 ^ab^	10.45	3.30	4.66 ^bc^	0.76 ^bcd^	0.24	0.12
		OP	54.2 ^ab^	37.3 ^ab^	10.91	3.42	4.91 ^abc^	0.78 ^bcd^	0.25	0.13
		MOS:OP	63.1 ^a^	43.7 ^a^	12.27	3.58	5.67 ^a^	1.01 ^ab^	0.31	0.15
		MOS:2OP	58.3 ^ab^	40.4 ^ab^	11.48	3.52	5.20 ^abc^	0.83 ^bcd^	0.26	0.13
		MOS:3OP	50.3 ^ab^	33.3 ^b^	10.50	3.18	5.04 ^abc^	1.04 ^a^	0.29	0.14
	HF	Control	49.5 ^b^	33.7 ^ab^	10.32	3.27	4.54 ^bc^	0.61 ^d^	0.23	0.12
		MOS	48.2 ^b^	32.8 ^b^	10.25	3.19	4.32 ^bc^	0.56 ^d^	0.22	0.11
		OP	46.0 ^b^	31.0 ^b^	9.87	3.15	4.24 ^c^	0.55 ^d^	0.21	0.11
		MOS:OP	52.4 ^ab^	35.8 ^ab^	10.68	3.35	4.82 ^abc^	0.74 ^bcd^	0.25	0.13
		MOS:2OP	58.7 ^ab^	40.7 ^sb^	11.56	3.54	5.26 ^ab^	0.80 ^bcd^	0.27	0.14
		MOS:3OP	51.1 ^ab^	34.3 ^ab^	10.55	3.25	4.95 ^abc^	0.89 ^abc^	0.29	0.14
		SEM	2.82	2.11	0.596	0.111	0.208	0.065	0.022	0.009
		*p* value	0.002	0.002	0.248	0.082	0.003	<0.001	0.040	0.097
		Pooled SEM	2.96	2.21	0.49	0.119	0.276	0.080	0.019	0.010
		Pooled *p* value								
		Diet	0.087	0.093	0.214	0.177	0.052	0.024	0.299	0.656
		Treatment	0.001	0.006	<0.001	0.293	<0.001	0.001	0.058	0.063
		Diet × Treatment	0.449	0.435	0.617	0.722	0.640	0.761	0.720	0.790

Within each incubation time, means in the same column with different superscripts differ, *p* < 0.05. *p*-value is the observed significance level of the F-test, SEM = standard error of the mean. ^1^ Diets were incubated without additives (Control treatment) or with the addition of 2% mannan oligosaccharide and onion peel combination at 1:0 (MOS treatment), 0:1 (OP treatment), 1:1 (MOS:OP treatment), 1:2 (MOS:2OP treatment) and 1:3 (MOS:3OP treatment). C_2_ is acetate, C_3_ is propionate, C_4_ is butyrate, C_5_ is valerate (mmol/g DM).

## Data Availability

Data are contained within the article.
